# Engineering biomarker representations of vital signs data enhances deep learning mortality prediction

**DOI:** 10.1093/jamia/ocag066

**Published:** 2026-05-02

**Authors:** Behrooz Mamandipoor, Isabella Shen, Chun-Nan Hsu, Rodney A Gabriel

**Affiliations:** Department of Biomedical Informatics, University of California, San Diego Health, La Jolla, CA, 92093, United States; Division of Perioperative Informatics, Department of Anesthesiology, University of California, San Diego, La Jolla, CA, 92037, United States; Division of Perioperative Informatics, Department of Anesthesiology, University of California, San Diego, La Jolla, CA, 92037, United States; Department of Biomedical Informatics, University of California, San Diego Health, La Jolla, CA, 92093, United States; Division of Perioperative Informatics, Department of Anesthesiology, University of California, San Diego, La Jolla, CA, 92037, United States

**Keywords:** deep learning, mortality, critical care medicine, predictive modeling, artificial intelligence

## Abstract

**Objectives:**

We evaluated bidirectional long short-term memory models for predicting inpatient mortality using different approaches to processing vital signs data collected during the initial 24 h of intensive care unit (ICU) admissions.

**Materials and Methods:**

We compared 3 vital-sign representations: (1) raw data recorded every 5 min, (2) preprocessed data averaged hourly, and (3) preprocessed data using biomarker representations that extends a digital oximetry biomarker toolbox of PhysioZoo software, applied to blood pressure, heart rate, temperature, respiratory rate, and SpO_2_.

**Results:**

Across 2 large ICU datasets, HiRID and eICU, models trained on the frequency-normalized representation achieved higher discrimination and lower Brier scores than those trained on raw 5-min and hourly averaged data.

**Discussion:**

The use of biomarker representations of vital signs yielded the largest improvements in discrimination and overall probabilistic performance reflected by lower Brier scores for predicting inpatient mortality by deep learning.

**Conclusion:**

Thus, we recommend using a similar approach to vital signs preprocessing for time-series predictive models.

## Introduction

Predicting critical care inpatient mortality using longitudinal vital sign time series remains a pressing challenge.^1^ Although intensive care units (ICUs) can monitor real-time patient vital sign trajectories, real-world vital sign data often contain missing values due to device malfunctions, inconsistent data collection schedules, and spurious measurements caused by artifacts. All of these elements contribute to excessive noise and irregularities in the data, potentially resulting in poor performance and generalizability of mortality prediction models built directly on raw s inputs.^2^

A key methodological gap is the lack of standardized preprocessing pipelines that transform heterogeneous bedside vital-sign streams into consistent representations suitable for predictive models. Therefore, adapting and validating existing computational pipelines for preprocessing time-series vital signs data is essential. PhysioZoo is a collaborative platform for continuous physiological time-series analysis in humans and other mammals,^3^ including a specific python toolbox for the study of digital oximetry biomarkers (Pulse Oximetry Benchmarking [POBM]).^4^^,^^5^ A prior study has demonstrated the utility of digital oximetry biomarkers derived from the open-source POBM toolbox. A biomarker-based oximetry model using engineered features by POBM toolbox outperformed a simpler oxygen desaturation index baseline in a multicenter study of polysomnography recordings across 6 independent databases.^4^ Compared with other platforms that offer universal features, it customizes analysis and feature selection to the specific physiological modality. However, by adjusting the analysis reasoning used in POBM, we can extend its implementation to other ICU vital signs (eg, blood pressure, temperature, respiratory rate, and heart rate). We hypothesized that extending this oximetry-focused framework could improve mortality prediction by normalizing sampling frequency and reducing noise while preserving clinically relevant dynamics.

In this study, we will determine whether a vital-sign biomarker generation pipeline can improve inpatient mortality prediction after the initial 24 h of ICU admission. Using deep learning time-series models, we compared 3 representations of the same first 24 h of vital-sign data: (1) raw data captured every 5 min, (2) nonoverlapping hourly averages of raw data, and (3) biomarker representations of sequence data using an extension of the POBM Dataset.

## Methods

### Data source

The study utilized deidentified publicly available data from the eICU Collaborative Research Database^6^^,^^7^ and the High-time Resolution ICU Dataset (HiRID),^8^^,^^9^ and was therefore exempt from the patient informed consent requirement by the Institutional Review Board (Human Research Protections Program). The eICU is a multicenter dataset specifically tailored for intensive care unit research, containing detailed, high-granularity data from over 200 859 ICU admissions involving 139 367 distinct patients across 335 units in 208 hospitals between 2014 and 2015 in the United States. The HiRID is a publicly accessible dataset that captures critical care data from more than 33 000 patients admitted to the Department of Intensive Care Medicine at the University Hospital of Bern, Switzerland, from January 2008 to June 2016.

### Outcome definition

The primary outcome was inpatient mortality occurring after the initial 24 h of the ICU admission (outcome was defined as death at any time in the hospital after this 24-h initial time period). To prevent information leakage, all data incorporated into the model were only those available within the initial 24-h observation window. As our prediction task was to predict mortality 24 h after ICU admission, ICU stays with a length of stay shorter than 24 h were excluded. Inclusion and exclusion criteria are outlined in Supplementary Material S1.

### Primary objective

**Figure 1.**
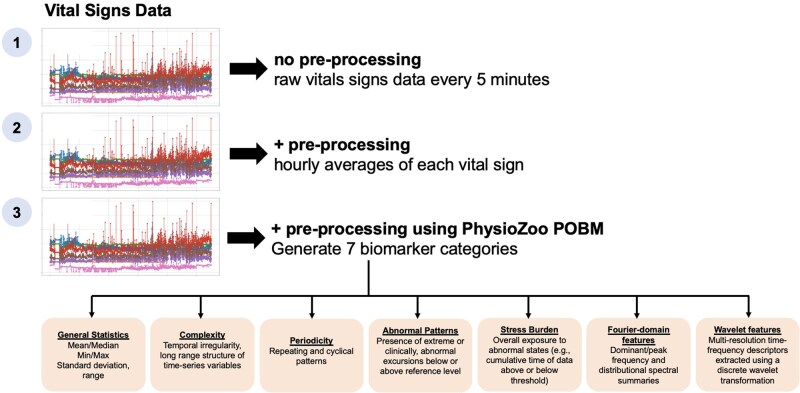
Using the same first 24-h observation window, we compared 3 approaches for representing vital signs data as model inputs: (1) raw data measured every 5 min without preprocessing; (2) preprocessing of data by using averaged hourly data, representing a common downsampling baseline; and (3) processed data using biomarkers generated by PhysioZoo POBM, which provides an engineered frequency normalization pipeline that transforms each vital sign variables into hourly feature vectors (7 different biomarker categories). Abbreviation: POBM, Pulse Oximetry Benchmarking Dataset.

The primary objective of this study was to develop time-series deep learning models for inpatient mortality prediction, incorporating high-frequency vital-sign data, and to quantify how different representations of vital sign time series affect predictive performance. Using the same first 24-h observation window, we compared 3 approaches (Figure 1) for representing vital signs data as model inputs: (1) raw data measured every 5 min without preprocessing; (2) preprocessing of data by using averaged hourly data, representing a common downsampling baseline; and (3) processed data using biomarkers generated by PhysioZoo POBM, which provides an engineered frequency normalization pipeline that transforms each vital sign variables into hourly feature vectors as described below.

### Vital-sign frequency normalization pipeline

Biomarkers for each vital sign were created by extending the PhysioZoo POBM to additional vitals, including heart rate, respiratory rate, temperature, and blood pressure (systolic/diastolic/mean). PhysioZoo was originally designed for analyzing continuous SpO_2_ time-series data and calculates specific biomarkers, including oxygen time-series distribution, long-range correlations, periodicity, and hypoxic burden. These biomarkers may then be used for downstream tasks. In this report, we apply this same biomarker processing with other vital signs, then apply those calculated biomarkers into our machine learning models to assess for performance improvement. PhysioZoo POBM is available as a Python toolbox (“pobm”).^10^ Starting from raw vital sign measurements in eICU and HiRID sampled uniformly as 5-min nonoverlapping time series, we extracted a fixed set of digital biomarkers using PhysioZoo POBM every hour using rolling windows of 3 h advanced by 1 h, yielding 1 feature vector per hour per signal. Within each rolling window, we computed 7 biomarker categories (Figure 1) described below. Supplementary Material S2 provides a more detailed and technical description on using PhysioZoo POBM in Python. Supplementary Material S3 reports the parameterization of the POBM pipeline, including the clinical thresholds specified for each vital sign and the parameters used for each biomarker category. The frequency of missing data for each vital sign in both datasets is also reported in Supplementary Material S4.

1. General statistics: Distribution and variability descriptors, including mean/median, minimum/maximum, SD, range, percentiles, below-median percentage, zero-crossings (signal crossing a baseline mean value), and a delta-index summarizing short-term variability.
2. Complexity: Measures of temporal irregularity and long-range structure of time-series variables, including approximate entropy and sample entropy (commonly used as measures of irregularity and unpredictability),^11^ along with complementary complexity descriptors (eg, Lempel-Ziv complexity, central tendency measure, and detrended fluctuation analysis).
3. Periodicity: Features capturing repeating/cyclical patterns, including autocorrelation-derived measures (correlation between values of the same variable over time at different lags), phase rectified signal averaging-based biomarkers, and power spectral density summaries over a clinically plausible low-frequency band.
4. Abnormal patterns: Event-based descriptors of extreme or clinically abnormal excursions below or above an adaptive reference level, providing information on their severity, duration, and frequency. For each window, low- and/or high-abnormal episodes were detected using quantile-based thresholds (eg, lower tail and upper tail cutoffs) and summarized event frequency and morphology (eg, duration, depth, area, slope, and time between events). This generalizes SpO_2_ desaturation logic to other analogs such as bradycardia and tachycardia in heart rate, and tachypnea and bradypnea in the respiratory rate.
5. Stress burden: Burden-style summaries quantify the overall exposure to abnormal states (extent and impact of abnormal vital sign levels on the body over time), including cumulative time and cumulative area below/above clinically defined thresholds (eg, hypoxic burden as SpO_2_ is below 92%, cardiac stress burden as heart rate outside a clinically prespecified range), providing integrated measures of physiologic stress.
6. Fourier-domain features: Fast Fourier transform- and short-time Fourier transform-derived spectral descriptors, including dominant/peak frequency and distributional spectral summaries (eg, centroid, bandwidth, flatness, roll-off, energy, and entropy).
7. Wavelet features: This captures localized oscillations and transient desaturations patterns of SpO_2_ using wavelet decomposition. Essentially, this computes wavelet biomarkers, such as wavelet energy at different scales, wavelet entropy, multiresolution variability, and time-frequency complexity measures.

### Model design and statistical analysis

We developed time-series models for predicting inpatient mortality using only vital signs data observed during the first 24 h after ICU admission. We trained a bidirectional long short-term memory (BiLSTM) classifier with a hidden size of 128 in each direction. The forward and backward outputs were concatenated and passed to a classification head consisting of a fully connected layer with 32 units, a rectified linear unit activation, and dropout, followed by a final output layer producing a mortality probability. To improve training stability and faster convergence, Xavier uniform initialization was applied to all linear and recurrent layers. To address class imbalance, we used weighted random sampling with probabilities inversely proportional to class frequencies, together with a weighted cross-entropy loss function. Models were optimized using Adam with a learning rate of 5 × 10^−4^ and weight decay of 0.01, with a batch size of 16. Training was stopped early if validation AUC or F1 score did not improve for 15 consecutive epochs, and the best-performing checkpoint on the validation set was retained. Hyperparameters were tuned based on validation-set performance.

We trained and evaluated models separately with the HiRID and eICU cohorts. For HiRID, we randomly split ICU stays into training/validation/test sets (70%/10%/20%). For eICU, we performed an institution-level data split for external validation, in which ICU stays from 8 specific institutions (each with more than 2500 ICU stays) were held out as the test set, and the remaining hospitals were used for training and validation. We compared models’ performance using the area under the receiver operating characteristic curve (AUROC), the area under the precision-recall curve (AUPRC), and the Brier score. To quantify uncertainty, we calculated 95% CI using the pivot bootstrap method with 1000 resamples of test sets.

## Results

### Study population

In the HiRID cohort, the final analytic sample included 16 642 patient stays, of which 1323 (7.9%) had an inpatient death occurring after the initial 24-h observation window. In the eICU, the final cohort included 132 837 patient stays, of which 6924 (5.2%) had an inpatient death after the first 24 h (Table 1).

**Table 1.** Basic demographic breakdown of survived and died cohort in the HiRID and eICU datasets.

	HiRID		eICU	
Variable	Survived	Died	Survived	Died
**Total**	15 319	1323	125 913	6924
**Male sex, *n* (%)**	9774 (63.8)	823 (62.2)	68 266 (54.2)	3807 (55.0)
**Age (years), median (quartiles)**	65.0 (55.0, 75.0)	70.0 (55.0, 75.0)	65.0 (53.0, 76.0)	69.0 (58.0, 79.0)

Abbreviations: eICU, electronic ICU Collaborative Research Database; HiRID, High-time Resolution Intensive Care Unit Dataset.

### Performance on the HiRID

In the HiRID cohort, the BiLSTM model using raw 5-min vital-sign sequences achieved an AUROC of 0.703 (95% CI, 0.665-0.739), an AUPRC of 0.249 (95% CI, 0.198-0.300), and a Brier score of 0.294 (95% CI, 0.287-0.300). Using hourly averaged vital signs reduced performance with AUROC, AUPRC, and Brier score of 0.680 (95% CI, 0.645-0.715), 0.212 (95% CI, 0.168-0.257), and 0.445 (95% CI, 0.439-0.452), respectively. In contrast, the biomarker representation using POBM substantially improved discrimination, achieving an AUROC of 0.852 (95% CI, 0.832-0.872), an AUPRC of 0.372 (95% CI, 0.313-0.432), and a Brier score of 0.190 (95% CI, 0.180-0.201) (Table 2).

**Table 2.** Performance metrics of each model on the test sets for HiRID and eICU.

	HiRID			eICU		
Model inputs	AUROC	AUPRC	Brier score	AUROC	AUPRC	Brier score
**Raw vital signs (every 5 min)**	0.703 (0.665-0.739)	0.249 (0.198-0.300)	0.294 (0.287-0.300)	0.746 (0.731-0.756)	0.246 (0.225-0.266)	0.274 (0.271-0.276)
**Raw vital signs (averaged per hour)**	0.680 (0.645-0.715)	0.212 (0.168-0.257)	0.445 (0.439-0.452)	0.736 (0.723-0.750)	0.236 (0.216-0.256)	0.297 (0.294-0.299)
**POBM processed vital signs**	0.852 (0.832-0.872)	0.372 (0.313-0.432)	0.190 (0.180-0.201)	0.836 (0.825-0.845)	0.331 (0.309-0.353)	0.211 (0.208-0.214)

Abbreviations: AUPRC, area under the precision-recall curve; AUROC, area under the receiver operating characteristics cruve; eICU, electronic ICU Collaborative Research Database; HiRID, High-time Resolution Intensive Care Unit Dataset; POBM, Pulse Oximetry Benchmarking Dataset.

### Performance on the eICU dataset

**Figure 2.**
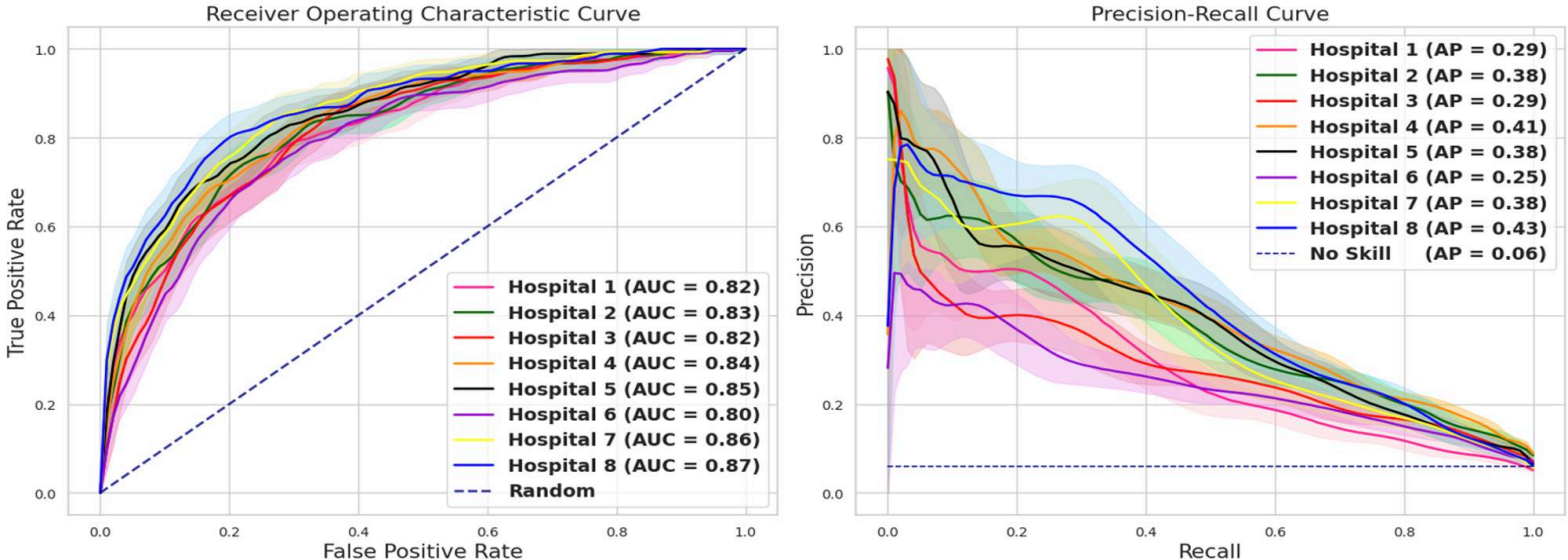
Model validation of 8 separate institutions from the Electronic Intensive Care Unit Collaborative Research Database. Abbreviations: AUC, area under the receiver operating characteristics curve; AP, average precision (area under the precision-recall curve).

In the eICU cohort, the BiLSTM model using raw 5-min vital-sign sequences achieved an AUROC of 0.746 (95% CI, 0.731-0.756), an AUPRC of 0.246 (95% CI, 0.225-0.266), and a Brier score of 0.274 (95% CI, 0.271-0.276). Using hourly averaged vital signs yielded similar discrimination with AUROC, AUPRC, and Brier score of 0.736 (95% CI, 0.723-0.750), 0.236 (95% CI, 0.216-0.256), and 0.297 (95% CI, 0.294-0.299), respectively. In contrast, the biomarker representation using POBM improved discrimination, achieving an AUROC of 0.836 (95% CI, 0.825-0.845), an AUPRC of 0.331 (95% CI, 0.309-0.353), and a Brier score of 0.211 (95% CI, 0.208-0.214) (Table 2). Furthermore, we calculated the model’s performance for each of the 8 institutions in the test set, with AUROC ranging from 0.80 to 0.87 (Figure 2). To assess potential overfitting, we additionally reported performance for each vital sign representation across training set and validation set in Supplementary Material S5.

## Discussion

Our study demonstrated that transforming longitudinal ICU vital signs into a digital biomarker representation using PhysioZoo POBM can improve the performance of a deep learning model for predicting mortality occurring after the first 24 h of ICU admission. Pulse Oximetry Benchmarking was originally designed for SpO_2_ data, but here we demonstrate how we can apply it to other vital signs, which downstream led to improve predictive modeling. We compared 3 representations of the same first 24 h of vital-sign data: (1) raw data recorded every 5 min, (2) nonoverlapping hourly averages of raw data, and (3) biomarker representations of vital signs using an extension of the PhysioZoo POBM toolbox.^3–5^

Using a raw 5-min time-series representation preserves high temporal resolution and captures fine-grained changes in a patient’s physiological state and short-term fluctuations. However, it may introduce noise, increase computational demands, and risk overfitting due to high dimensionality and data volume. In contrast, hourly averaging simplifies the data, reduces variance and noise, and lowers computational requirements, but may remove clinically relevant temporal information and smooth out critical short-term fluctuations essential for accurate predictions. To balance these tradeoffs, we implemented a vital-sign frequency normalization pipeline by extending the feature engineering methods of the open-source POBM toolbox^4^^,^^5^ in the PhysioZoo software.^3^ POBM focuses on the physiological interpretation and clinical use of oximetry biomarkers, which can be adapted to different physiological data modalities as they share similar structures. PhysioZoo has already released platforms for heart rate variability and electrocardiography biomarkers, demonstrating the broad applicability of the framework.

While POBM is typically applied solely to SpO_2_, we extended its application to process other ICU vital signs including blood pressure, heart rate, temperature, and respiratory rate. Within rolling multihour windows advanced at an hourly stride, we computed 7 feature categories: general statistics, complexity, periodicity, abnormal-pattern descriptors, stress-burden measures, and complementary frequency-domain and time-frequency descriptors (Fourier transform and wavelet-based). This approach standardizes heterogeneous bedside streams into a consistent longitudinal feature table, reduces sensitivity to measurement noise, and preserves clinically meaningful dynamics that are not captured by simple averages. In our study, the frequency-normalized representation of vital signs yielded the largest improvements in discrimination for predicting inpatient mortality by deep learning. Thus, we recommend using a similar approach to vital signs preprocessing for time-series predictive models.

Several limitations merit discussion: First, raw measurements were available at 5-min resolution, which restricts detection of very rapid physiologic events (eg, transient arrhythmias or short blood pressure spikes) occurring between samples. Second, some event-based biomarkers were initially developed for higher frequency oximetry, and when applied to coarser sampling, their parameterization may underrepresent short events and alter estimates of event morphology. Third, this study did not compare the extended POBM-based representation with alternative methods for generating digital biomarkers. Therefore, although it outperformed the baselines examined here, future work should directly compare this approach with other engineered and learned representations of physiological time-series data. Future studies should also evaluate this pipeline in datasets with higher sampling frequencies and use ablation studies, redundancy analysis, modality-specific feature selection, and interpretability methods to identify the biomarker categories that are most informative and generalizable for representing ICU vital-sign data. Another important direction is to refine the definitions used in threshold-based biomarker categories, particularly stress-burden features, as the most informative thresholds may vary across modalities, datasets, and prediction tasks. This question could be examined through threshold tuning guided by domain knowledge, sensitivity analyses, and data-driven optimization. Despite these constraints, our findings support using engineered digital biomarkers as a practical and generalizable strategy for improving predictive modeling with routinely collected ICU vital signs time series. This is essential for future clinical applications given the reality of consistent data missingness and noise in electronic health data. Our study demonstrates how to handle this type of data, which may lead to improved clinical models.

## Supplementary Material

ocag066_Supplementary_Data

## Data Availability

Data are made available from the eICU (https://physionet.org/content/eicu-crd/2.0/) and HiRID (https://physionet.org/content/hirid/1.1.1/) public repositories after appropriate data use agreements in place.
