# Supplementary material for: Engineering biomarker representations of vital signs data enhances deep learning mortality prediction
Source: J Am Med Inform Assoc. 2026 May 2;33(7):1381–6. doi: 10.1093/jamia/ocag066 (PMC13317957; doi:10.1093/jamia/ocag066)
Supplement: ocag066_Supplementary_Data [file ocag066_supplementary_data.zip › Supplemental File 4.docx]

**Supplemental File 4**

Missing values were handled using a two-step imputation strategy. First, observed values were forward-filled for up to 12 hours to preserve recently measured physiologic information. Second, any remaining missing values were imputed using the mean value of the corresponding vital sign calculated from the training data.

In the HiRID dataset, heart rate had the lowest imputation rate at 4.7% of observations (median per patient: 0.4%), followed by SpO₂ at 7.6% (median: 3.5%) and systolic, diastolic, and mean blood pressure at approximately 10.8% each (median: 3.1%). Respiratory rate required imputation in 46.2% of observations (median: 37.2%), while temperature had the highest imputation rate at 84% (median: 98%), consistent with its intermittent manual recording.

In the eICU dataset, heart rate had the lowest imputation rate at 7.0% (median per patient: 1.7%), followed by SpO₂ at 15.2% (median: 5.6%) and respiratory rate at 17.4% (median: 4.2%). Blood pressure variables had imputation rates of approximately 82% each (median: 84%), and temperature was imputed in 90% of observations (median: 97%). These patterns reflect differences in bedside measurement practices, including intermittent manual recording of temperature and less frequent blood pressure measurements in eICU.
