# Supplementary material for: Engineering biomarker representations of vital signs data enhances deep learning mortality prediction
Source: J Am Med Inform Assoc. 2026 May 2;33(7):1381–6. doi: 10.1093/jamia/ocag066 (PMC13317957; doi:10.1093/jamia/ocag066)
Supplement: ocag066_Supplementary_Data [file ocag066_supplementary_data.zip › Supplemental File 5.docx]

**Supplemental File 5**

To assess potential overfitting, we additionally computed performance for each vital signs representation in training-set and validation-set.

| **HiRID** | **Training-set** | | | **Validation-set** | | |
| --- | --- | --- | --- | --- | --- | --- |
| **Model Inputs** | **AUROC** | **AUPRC** | **Brier Score** | **AUROC** | **AUPRC** | **Brier Score** |
| Raw vitals signs (every 5 minutes) | 0.712 | 0.245 | 0.296 | 0.737 | 0.260 | 0.298 |
| Raw vitals signs (averaged per hour) | 0.702 | 0.237 | 0.434 | 0.705 | 0.217 | 0.433 |
| POBM processed vital signs | 0.903 | 0.400 | 0.164 | 0.846 | 0.336 | 0.185 |

| **eICU** | **Training-set** | | | **Validation-set** | | |
| --- | --- | --- | --- | --- | --- | --- |
| **Model Inputs** | **AUROC** | **AUPRC** | **Brier Score** | **AUROC** | **AUPRC** | **Brier Score** |
| Raw vitals signs (every 5 minutes) | 0.773 | 0.225 | 0.274 | 0.769 | 0.213 | 0.275 |
| Raw vitals signs (averaged per hour) | 0.772 | 0.215 | 0.300 | 0.759 | 0.205 | 0.302 |
| POBM processed vital signs | 0.866 | 0.322 | 0.177 | 0.848 | 0.307 | 0.179 |
