# Supplementary material for: Engineering biomarker representations of vital signs data enhances deep learning mortality prediction
Source: J Am Med Inform Assoc. 2026 May 2;33(7):1381–6. doi: 10.1093/jamia/ocag066 (PMC13317957; doi:10.1093/jamia/ocag066)
Supplement: ocag066_Supplementary_Data [file ocag066_supplementary_data.zip › Supplemental File 2.docx]

**Supplemental File 2**

PhysioZoo was originally designed for analyzing continuous SpO_2_ data time series data and calculates specific biomarkers, including oxygen time series distribution, long range correlations, periodicity, and hypoxic burden^1,2^ . These biomarkers may then be used for downstream tasks. We apply this same biomarker processing with other vital signs, then apply those calculated biomarkers into our machine learning models to assess for performance improvement.

PhysioZoo POBM is available as a Python toolbox (pobm). Once imported into Python, the user can upload a 1D array of time series data for a specific vital sign (e.g., SpO_2_) and sampling rate frequency. Within the POBM package, there are various biomarkers grouped into categories that the user may compute with the data. The following are example biomarker categories that were leveraged for our study and their associated python functions with the pobm python package:

1) **General statistics** (python package = OverallGeneralMeasures): distribution and variability descriptors, including mean/median, minimum/maximum, standard deviation, range, percentiles, below-median percentage, zero-crossings (signal crossing a baseline mean value), and a delta-index summarizing short-term variability.

2) **Complexity** (python package = ComplexityMeasures): measures of temporal irregularity and long-range structure of time-series variables, including approximate entropy and sample entropy (commonly used as measures of irregularity and unpredictability), along with complementary complexity descriptors (e.g., Lempel–Ziv complexity, central tendency measure, and detrended fluctuation analysis).

3) **Periodicity** (python package = PRSAMeasures): features capturing repeating/cyclical patterns, including autocorrelation-derived measures (correlation between values of the same variable over time at different lags), Phase rectified signal averaging (PRSA)-based biomarkers, and power spectral density summaries over a clinically plausible low-frequency band. Low and high thresholds can be set in this function.

4) **Abnormal patterns** (python package = DesaturationMeasures): event-based descriptors of extreme or clinically abnormal excursions below or above an adaptive reference level, providing information on their severity, duration, and frequency. For each window, low- and/or high-abnormal episodes were detected using quantile-based thresholds (e.g., lower-tail and upper-tail cutoffs) and summarized event frequency and morphology (e.g., duration, depth, area, slope, and time between events). This generalizes SpO₂ desaturation logic to other analogs such as bradycardia and tachycardia in heart rate, and tachypnea and bradypnea in the respiratory rate.

5) **Stress burden** (python package = HypoxicBurdenMeasures): burden-style summaries quantify the overall exposure to abnormal states (extent and impact of abnormal vital sign levels on the body over time), including cumulative time and cumulative area below/above clinically defined thresholds (e.g., hypoxic burden as SpO₂ is below 92%, cardiac stress burden as heart rate outside a clinically prespecified range), providing integrated measures of physiologic stress. The user can set the thresholds of what is normal using this function.

6) **Fourier-domain features** (python package = PSDMeasures): Fast Fourier Transform (FFT) and Short-Time Fourier Transform (STFT) derived spectral descriptors, including dominant/peak frequency and distributional spectral summaries (e.g., centroid, bandwidth, flatness, roll-off, energy, and entropy).

7) **Wavelet features** (python package = WaveletMeasures): multi-resolution time–frequency descriptors extracted using a discrete wavelet transform (Daubechies-4; level 2) to capture both time and frequency domain characteristics, summarizing approximation and detail coefficients via statistics (e.g., mean/variance/SD/skewness/kurtosis) and energy/entropy features. It captures localized oscillations and transient desaturation patterns of SpO_2_ using wavelet decomposition. Essentially, this computes wavelet biomarkers, such as wavelet energy at different scales, wavelet entropy, multi-resolution variability, and time-frequency complexity measures.

Each of these biomarkers were calculated and then included as inputs for downstream classifier models. We compared models predicting mortality using three different approaches to feature representation: (1) no pre-processing (raw data every 5 minutes); (2) pre-processed data by averaging hourly values; and (3) pre-processed data using these biomarkers from PhysioZoo POBM.

**References**

1. Levy J, Alvarez D, Del Campo F, Behar JA. Deep learning for obstructive sleep apnea diagnosis based on single channel oximetry. Nat Commun 2023;14:4881.

2. Levy J, Alvarez D, Rosenberg AA, Alexandrovich A, Del Campo F, Behar JA. Digital oximetry biomarkers for assessing respiratory function: standards of measurement, physiological interpretation, and clinical use. NPJ Digit Med 2021;4:1.
