# Supplementary material for: Engineering biomarker representations of vital signs data enhances deep learning mortality prediction
Source: J Am Med Inform Assoc. 2026 May 2;33(7):1381–6. doi: 10.1093/jamia/ocag066 (PMC13317957; doi:10.1093/jamia/ocag066)
Supplement: ocag066_Supplementary_Data [file ocag066_supplementary_data.zip › Supplemental File 3.docx]

**Supplemental File 3**

This section details the specific parameters and clinically defined thresholds used to configure the PhysioZoo POBM pipeline for each vital sign in this study.

First, clinically defined fixed thresholds were specified for signal-specific configuration in burden-style summaries. For SpO₂, only lower-bound abnormal states were considered, with a reference threshold of 92%. For temperature, only upper-bound abnormal states were considered, with a threshold of 38°C. For heart rate, lower- and upper-bound reference thresholds were 70 and 110 beats/min, respectively. For respiratory rate, thresholds were 12 and 18 breaths/min. For systolic blood pressure, thresholds were 95 and 125 mmHg; for diastolic blood pressure, 75 and 85 mmHg; and for mean arterial pressure, 85 and 105 mmHg. These fixed thresholds were used to define clinically interpretable burden measures and to provide modality-specific reference ranges. Second, abnormal-pattern episodes were identified within each rolling window using a quantile-based adaptive detection strategy, rather than fixed clinical cutoffs. Specifically, the 15th percentile of the windowed signal was used to detect lower-bound abnormal episodes and the 85th percentile was used to detect upper-bound abnormal episodes.

For the general statistics biomarkers, the zero-crossing baseline was set to the signal mean, the below-median threshold was set to 2, and the delta-index window was set to 12. Periodicity biomarkers were computed using a phase-rectified signal averaging (PRSA) window equal to one-third of the rolling window length and an autocorrelation lag of 2. Power spectral density features were calculated over a fixed low-frequency band defined in the implementation (0.003–0.042 in the pipeline frequency units). Complexity biomarkers were computed using a central tendency measure radius of 2, a detrended fluctuation analysis window equal to 12, sample entropy parameters of embedding dimension 2 and tolerance 0.2, and approximate entropy parameters of embedding dimension 2 and tolerance 0.2. Stress-burden measures used the vital-sign-specific clinical thresholds described above, with the cumulative area baseline set to the signal mean. Fourier-domain features were computed over the full rolling window length of 36 samples, and wavelet biomarkers were extracted using a Daubechies-4 wavelet at decomposition level 2.
