# Supplementary figures and images for: Engineering biomarker representations of vital signs data enhances deep learning mortality prediction
Source: J Am Med Inform Assoc. 2026 May 2;33(7):1381–6. doi: 10.1093/jamia/ocag066 (PMC13317957; doi:10.1093/jamia/ocag066)

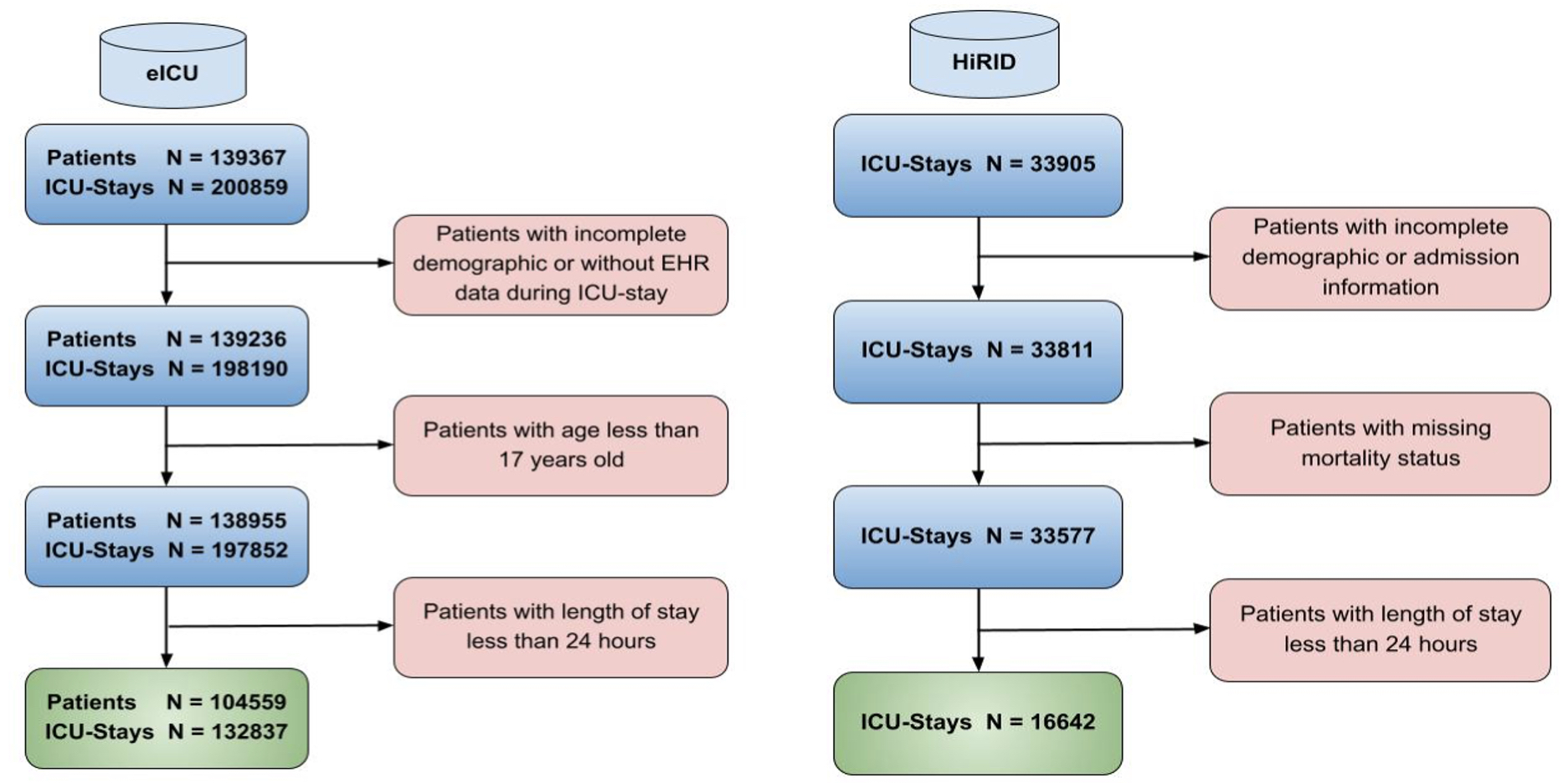

Supplement: ocag066_Supplementary_Data [file ocag066_supplementary_data.zip › Supplemental File 1.jpg]
